# Financial buy-in does not affect outcomes of endoscopic sleeve gastroplasty: Retrospective cohort

**DOI:** 10.1055/a-2631-7439

**Published:** 2025-07-23

**Authors:** Lea Sayegh, Karl Akiki, Karim Al Annan, Yara Salameh, Khushboo Gala, Kamal Abi Mosleh, Manpreet Mundi, Omar Ghanem, Barham K. Abu Dayyeh, Andrew C. Storm

**Affiliations:** 1 4352 Division of Gastroenterology and Hepatology, Mayo Clinic, Rochester, United States; 2 6915 Surgery, Mayo Clinic, Rochester, United States; 3 Endocrinology, Mayo Clinic, Rochester, United States

**Keywords:** Epidemiology

## Abstract

**Background and study aims:**

Endoscopic sleeve gastroplasty (ESG) is an effective treatment for obesity but typically is not covered by insurance. It is not known whether patients with financial investment in their endoscopic procedure are more likely to achieve and/or maintain weight loss as compared with those who have no financial buy-in. We aimed to compare treatment adherence and outcomes between patients paying out-of-pocket (OOP) and those who underwent ESG as part of any clinical trial where costs were covered by a study protocol (no payment; NP).

**Patients and methods:**

Data were collected via retrospective chart review. One hundred sixty-four patients who had an ESG with at least 6 months of follow-up were included. Repeated measures with generalized linear model were used to evaluate weight loss at different time points after ESG and labs values at baseline and 1-year follow-up to assess for comorbidity improvement between cohorts. Compliance was evaluated by comparing exercise adherence rates.

**Results:**

The pattern of weight loss and change in laboratory values was not different over time between the OOP group (n = 139) and NP group (n = 25). Patients lost an average of 14% (12.2–15.9) and 12.9% (9.3–16.5) of total body weight over all time points, respectively, in both groups (6, 12 and 24 months). Treatment adherence also did not differ between the groups.

**Conclusions:**

Having “skin in the game” by paying for ESG OOP does not correlate with better outcomes or treatment adherence, which further supports broad insurance coverage for this procedure.

## Introduction

Endoscopic sleeve gastroplasty (ESG) is an established endobariatric technique that uses an endoscopic suturing device to remodel and reduce stomach volume 1 . Clinical trials such as the MERIT study 2 have proven its superior efficacy compared with lifestyle modifications alone and highlight the favorable safety profile of this non-surgical weight loss procedure. ESG is associated with a lower adverse event rate as compared with laparoscopic sleeve gastrectomy 3 and it may be the only viable option for medically complex patients or patients with moderate (Class I) obesity, because both groups often do not qualify for weight loss surgery 1 .

ESG is not yet routinely covered by insurance in the United States, and therefore, patients most often either pay for the procedure out of pocket (OOP) or may rarely enroll in a clinical trial where the cost of the procedure is covered by research protocol funding 3 . The financial burden of ESG may be a factor limiting accessing to this minimally invasive tool for weight loss in many potential candidates, because obesity has been associated with lower socioeconomic status 4 . Societal norms have often suggested that management of obesity is a personal responsibility and that insuring coverage of costs associated with obesity treatment will lead to inferior adherence to treatments 5 . This paradigm was refuted by Ard et. al who found no difference in treatment outcomes when comparing covered patients with self-payers who underwent medical weight loss treatment. Conversely, they described lower levels of attrition among covered patients 5 .

Whether having financial “skin in the game” results in better outcomes or adherence to treatment after a weight loss procedure like ESG has not yet been described. We hypothesized that patients who pay for an ESG weight loss procedure do not have significantly improved outcomes or treatment adherence as compared with patients whose procedure has no associated out of pocket costs.

## Patients and methods

### Study design

This was a retrospective review of a prospectively collected database conducted at a tertiary care center. Two hundred forty-five patients who had undergone ESG were identified, of whom 164 had follow-up for at least 6 months after the procedure and were included in the analysis.

### Study sample

Patients were divided into two groups: the no payment (NP) group for those who had an ESG performed in the context of a funded trial, and the OOP group for all other ESG patients who paid for the procedure OOP. No separate analysis was done to compare men and women among groups, due to the small sample size and difficulty in extracting meaningful conclusions. Factors related to income and socioeconomic status were not considered.

### Collected variables

Variables relating to demographics, anthropometrics, comorbidity-related laboratory values, and medication intake were extracted from patient charts.

## Ethics considerations

The study was performed according to the Declaration of Helsinki. All procedures performed in studies involving human participants were in accordance with the ethical standards of the institutional and/or national research committee and with the 1964 Helsinki declaration and its later amendments or comparable ethical standards.

The study was approved and deemed exempt by our institutional review board on October 31, 2022, ID number 22–010115.

### Outcomes

#### Primary outcome

Weight loss was assessed using % total body weight loss (%TBWL) at 6 (± 2 months), 12 (± 3 months) and 24 months (± 3 months) after the procedure. To avoid confusion, it was reported as %TBWC, and was calculated using the standard definition, namely %TBWC = (follow-up weight – baseline weight)/ (baseline weight) X 100%. %TBWC was reported for the total sample and compared between the OOP and NP groups at all time points. When negative, the value represents weight loss; when positive, weight gain. Absolute weight was also compared within and between groups in repeated measures generalized linear model (GLM) analysis.

#### Secondary outcomes

Comorbidity improvements were assessed using relevant serum laboratory values, specifically fasting glucose (FG) and HbA1c for diabetes and low-density lipoprotein and TG for hyperlipidemia. Differences between baseline and follow-up values at 1 year (± 3 months) were compared between OOP and NP groups using repeated measure with GLM to compare patterns of changes in these values over time and between groups.

Treatment adherence was measured by assessing self-reported adherence to an exercise regimen 1 year after ESG on follow up. This was assessed by evaluating clinic notes. Patients who reported exercising most days, with a mix of endurance and strength training, were considered adherent. Those who reported no exercise or occasional walking were considered non-adherent.

### Statistical analysis

Considering the small sample size of one of the groups, continuous variables were assessed and were described as medians (IQR [interquartile range]). Categorical variables were described as proportions or percentages. When not specified, values presented as x (y+z) represent a median and IQR in brackets. Ninety-five percent confidence intervals (CIs) will be specified as such. Significance level was set at 0.05. Considering the small group size of the NP group, repeated measures with GLM with identity link was used to compare both groups across different time points in terms of weight loss and laboratory value changes for all four parameters. The between-subject factor was participation in a clinical trial (trial vs. no trial), whereas time was treated as the within-subject variable. An unstructured working correlation matrix was used to allow flexible correlations between time points.

## Results

### Baseline characteristics

One hundred sixty-four patients who underwent ESG and had follow-up for at least 6 months after the procedure were identified and included in the analysis. For the OOP group, median age was 50.7 (42.0–59.1) and 79.9% of patients were female ( Table 1 ). At baseline, 98 participants (70.5%) had at least one comorbidity with the most prevalent comorbidity being HLD (46.8%), whereas the least prevalent was T2DM (7.9%). Seventy-seven participants (55.4%) were taking at least one comorbidity-related medication (AOM). Median baseline body mass index (BMI) was 36.1 (33.9–40.3). Six participants (4.3%) were smokers and 19 (11.6%) took anti-obesity medication (AOM).

**Table:** **Table 1** Baseline characteristics for OOP and NP patients.

Mean± SD/median (Q1-Q3)/N (%)	OOP (n = 139)	NP (n = 25)
Women	111 (79.9)	20 (80.0)
Age	50.7 (41.2–59.1)	54.1 (43.0–56.9)
HTN (yes)	54 (38.8)	15 (60.0)
T2DM (yes)	11 (7.9)	7 (28.0)
HLD (yes)	65 (46.8)	12 (48.0)
Psychiatric disorder(s) (yes)	45 (32.4)	9 (36.0)
Taking medication for HTN (yes)	50 (36.0)	13 (52.0)
Taking medication for T2DM (yes)	12 (8.6)	6 (24.0)
Taking medication for HLD (yes)	31 (22.3)	8 (32.0)
Taking medication for psychiatric disorders (yes)	49 (35.3)	10 (40)
HTN on medication (≥ 2)	19 (37.3)	5 (33.3)
T2DM on medication (≥ 2 meds)	4 (40.0)	3 (42.9)
T2DM on GLP-1 Medications	0 (0)	1 (14.3)
HLD on medication: (yes)	26 (40.0)	7 (58.3)
Psychiatric disorders on medication (≥ 2 meds)	23 (56.1)	3 (33.3)
Baseline weight (kg)	100.0 (91.1–117.0)	99.9 (90.5–107.0)
Baseline BMI (kg/m ^2^ )	36.1 (33.9–40.3)	35.8 (32.7–37.5)
Baseline FG (mg/dL) (n = 110 and 25)	101.5 (92.0–111.0)	98.0 (92.0–117.8)
Baseline LDL (mg/dL) (n = 114 and 25)	108.4 ± 33.6	111.1 ± 44.0
Baseline TGs (mg/dL) (n = 116 and 25)	134.0 (103.3–191.0)	127.0 (81.5–157.5)
Baseline HbA1c (%) (n = 53 and 25)	5.6 (5.2–6.1)	5.4 (5.3–6.7)
Smoking (yes)	6 (4.3)	0 (0)
AOMs (yes)	19 (13.7)	1(4)
AOM, anti-obesity medication; FG, fasting glucose; Hba1c, hemoglobin A1c; HLD, hyperlipidemia; LDL, low-density lipoprotein; Med, medication; NP, no payment; OOP, out of pocket; Q1, quartile 1; Q3, quartile 3; SD, standard deviation; T2DM, type 2 diabetes mellitus; TG, triglyceride.		

For the NP group, median age was 54.1 (43.0–56.9) and 80.0% of patients were female ( Table 1 ). At baseline, 21 participants (84.0%) had at least one comorbidity with the most prevalent comorbidity being HTN (60.0%), whereas the least prevalent was T2DM (28.0%). Seventeen participants (68.0%) were taking at least one comorbidity-related medication. Median baseline BMI was 35.8 (32.7–37.5). Six participants (4.3%) were smokers and one (4.0%) took AOM.

Baseline characteristics did not appear markedly different between OOP and NP groups regarding most variables except for T2DM, with OOP patients having a lower rate of diabetes, 7.9% versus 28.0%.

Baseline characteristics were also described between patients who had a weight recorded at 6 months, 12 months, and 24 months and those who did not (i.e., dropouts at each stage), and are detailed in **Supplementary Table 1** , **Supplementary Table 2** , and **Supplementary Table 3** , respectively. Only hypertension at baseline appeared different between groups at 6 months, with a higher proportion of patients in the dropout group having hypertension, although this difference narrowed at 12 months and 24 months.

**Fig. 1:**
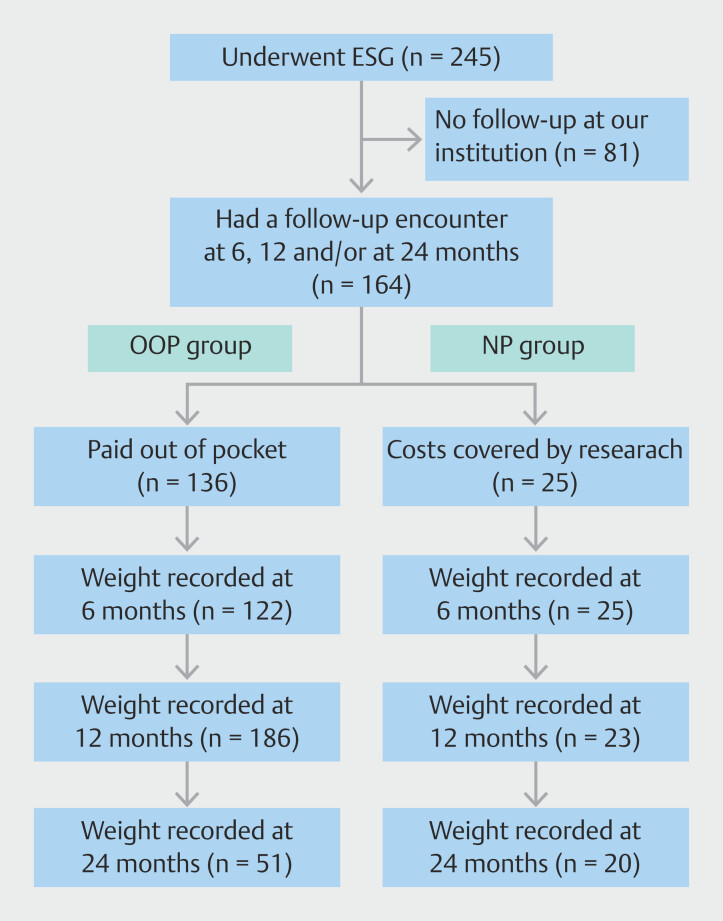
Flowchart showing patients who had weights recorded at 6, 21 and 24 months among both OOP and NP groups.

To illustrate follow-up, a flowchart was constructed to show population samples at different time points ( Fig. 1 ).

### Weight loss

%TBWL at 6, 12, and 24 months expressed as median with IQR was 13.9 (9.6–18.9), 12.4 (7.4–19.9), and 10.7 (5.2–19.2) for the OOP group and 17.0 (11.4–19.3), 15.8 (9.2–22.1), and 10.3 (5.7–18.3) for the NP group, respectively.

There was no significant difference in between-group means in terms of weight at baseline, 6 months, 12 months, or 24 months. In terms of %TBWC, there were no significant differences at all time points as well ( Table 2 , Table 3 , Table 4 , Table 5 , Table 6 ). %TBWC was also similar between group across all time points.

**Table:** **Table 2** Mean weight (kg), TBWC (%), FG (mg/dL), TG (mg/dL), LDL (mg/dL), and HbA1c with corresponding 95% confidence intervals (CIs) at baseline.

Measure	N OOP group	N NP group	Total N	Mean OOP group (95% CI)	Mean NP group (95% CI)	Between group difference mean (95% CI)
Weight	139	25	164	104.8 (101.3, 108.3)	101.2 (96.5–105.9)	–3.7 (–8.3, 1.0)
FG	110	25	135	107.2 (102.1, 112.3)	112.2 (98.9, 125.9)	5.0 (–9.5, 19.5)
LDL	114	25	139	110.3 (104.1, 116.4)	110.4 (93.8, 127.0)	0.11 (–17.6, 17.8)
TG	116	25	141	148.9 (137.1, 160.8)	128.4 (106.0, 150.8)	20.6 (–45.9, 4.8)
HbA1c	53	25	78	5.7 (5.5, 5.9)	5.8 (5.2, 5.6)	–0.27 (–1.5, 1.0)
Represented is also between-group mean differences and their CIs, within-group differences at each time point compared to baseline. Note that reference groups for time and group are baseline time and OOP group, respectively. FG, fasting glucose; HbA1c, hemoglobin A1c; LDL, low-density lipoprotein; NP, no payment; OOP, out of pocket; TBWC, total body weight loss; TG, triglyceride.						

**Table:** **Table 3** Mean weight (kg) and TBWC (%).

Measure	N OOP group	N NP group	Total N	Mean OOP group (95% CI)	Mean NP group (95% CI)	Mean difference in overall sample between baseline and 6 months (95% CI)	Between Group difference Mean (95% CI)
Weight	122	25	147	89.3 (86.1 to 92.4)	85.5 (81.4 to 89.6)	–15.6 (–17.2 to -14.0)	–3.8 (–8.7–1.1)
TBWC	122	25	147	–14.5 (–15.7 to –13.4)	–15.3 (–17.8 to -12.9)	–14.9 (–17.3 to –12.6)	–0.8 (–3.5 to –1.9)
Represented is also between-group mean differences and their CIs, within-group differences at each time point compared to baseline. Fig. 2 represents overall within-group differences across all time points for OOP and NP groups, overall between groups differences across all time points, all with 95% CIs. NP, no payment; OOP, out of pocket; TBWC, total body weight loss.							

**Fig. 2:**
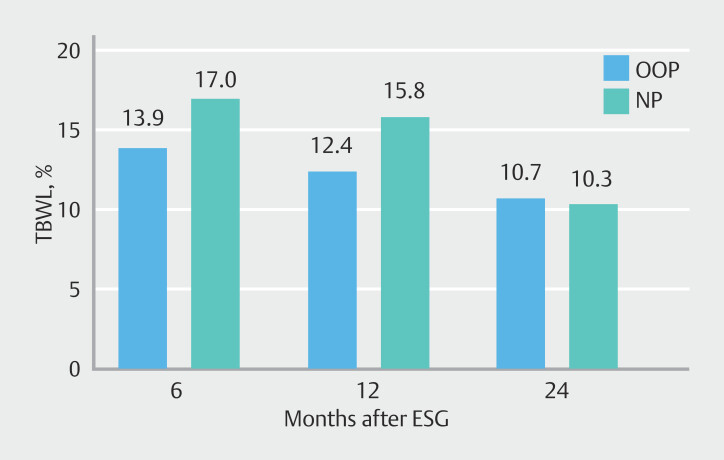
%TBWL at 6, 12 and 24 months for out-of-pocket (OOP) and no payment (NP) groups.

**Table:** **Table 4** Mean weight (kg), TBWC (%), FG (mg/dL), TG (mg/dL), LDL (mg/dL), and HbA1c with corresponding 95% CIs at 12 months.

Measure	N OOP group	N NP group	Total N	Mean OOP group (95% CI)	Mean NP group (95% CI)	Mean difference in overall sample between baseline and 12 months (95% CI)	Between group difference mean (95% CI)
Weight	86	23	109	89.7 (86.4–93.1)	86.2 (81.5–91.0)	–15.1 (–17.4 to–12.9)	–3.5 (–9.4–2.4)
TBWC	86	23	109	–14.2 (–15.9 to –12.5)	–13.8 (–17.5 to –10.1)	–14.0 (–16.9 to –11.1)	0.4 (–3.7–4.5)
FG	77	24	101	98.4 (94.5–p 102.3)	101.2 (93.7–108.6)	–10.0 (–18.4 to –1.7)	2.8 (–5.6–11.2)
LDL	83	25	108	99.8 (93.3–106.3)	113.9 (97.0–130.8)	–3.5 (–16.2–9.2)	14.1 (–4.0–32.3)
TG	83	25	108	126.6 (115.7–137.4)	109.2 (92.6–125.7)	123.3 (112.4–134.2)	–17.4 (–37.2–2.4)
HbA1c	68	23	91	5.4 (5.3–5.6)	5.4 (5.2–5.6)	0.03 (–1.6–1.7)	–0.4 (–2.4–1.5)
Represented is also between-group mean differences and their CIs, within-group differences at each time point compared to baseline. CI, confidence interval; FG, fasting glucose; HbA1c, hemoglobin A1c; LDL, low-density lipoprotein; NP, no payment; OOP, out of pocket; TG, triglyceride.							

**Table:** **Table 5** Mean weight (kg) and TBWC with corresponding 95% CIs at 24 months.

Measure	N OOP group	N NP group	Total N	Mean OOP group (95% CI)	Mean NP group (95% CI)	Mean difference in overall sample between baseline and 24 months (95% CI)	Between group difference mean (95% CI)
Weight	51	20	71	89.1 (84.0–94.2)	94.1 (86.7–101.5)	–15.7 (–19.9 to -11.5)	5.0 (–4.0–14.0)
TBWC	51	20	71	–13.4 (–15.8 to –10.9)	–9.6 (–13.9 to -5.3)	–11.5 (–15.0 to -8.0)	3.8 (–1.1–8.7)
Represented is also between-group mean differences and their CIs, within-group differences at each time point compared to baseline. CI, confidence interval; NP, no payment; OOP, out of pocket; TBWC, total body weight loss.							

**Table:** **Table 6** Overall within-group differences across all time points for OOP and NP groups, all with 95% CIs.

Measure	Within-group mean difference for OOP group (95% CI)	Within-group mean difference for NP group (95% CI)	Overall between-group mean difference (95% CI)
Weight	–15.5 (–18.5 to –12.5)	–14.0 (–16.57 to –11.47)	1.5 (–4.3–7.2)
TBWC	–14.0 (–15.9 to –12.2)	–12.9 (–16.5 to –9.3)	0.8 (–1.5–3.2)
FG	–8.8 (–15.2 to –2.4)	–11 (–26.4–4.4)	4.0 (–5.1–13.1)
LDL	–10.5 (–28.2–7.2)	3.5 (–20.2–27.2)	7.1 (–9.8–24.0)
TG	–22.4 (–38.4 to –6.3)	–19.2 (–47.1–8.7)	–19.0 (–39.2–1.2)
HbA1c	0.1 (–0.3–0.5)	–0.05 (–2.3–2.2)	–0.3 (–1.9–1.3)
Represented is also between-group mean differences and their CIs, within-group differences at each time point compared to baseline. CI, confidence interval; FG, fasting glucose; HbA1c, hemoglobin A1c; LDL, low-density lipoprotein; NP, no payment; OOP, out of pocket; TBWC, total body weight loss; TG, triglyceride.			

Within-group differences across time were also similar for both groups, with the OOP group losing on average 14% of body weight over time (12.2–15.9) vs 12.9% for the NP group (9.3–16.5).

In summary, although there was a significant decrease in weight over time across the entire sample, there were no significant differences in weight changes between participants in clinical trials and those not in trials, nor was there a significant difference in rate of weight change over time between the two groups.

### Comorbidity improvement

**Fig. 3:**
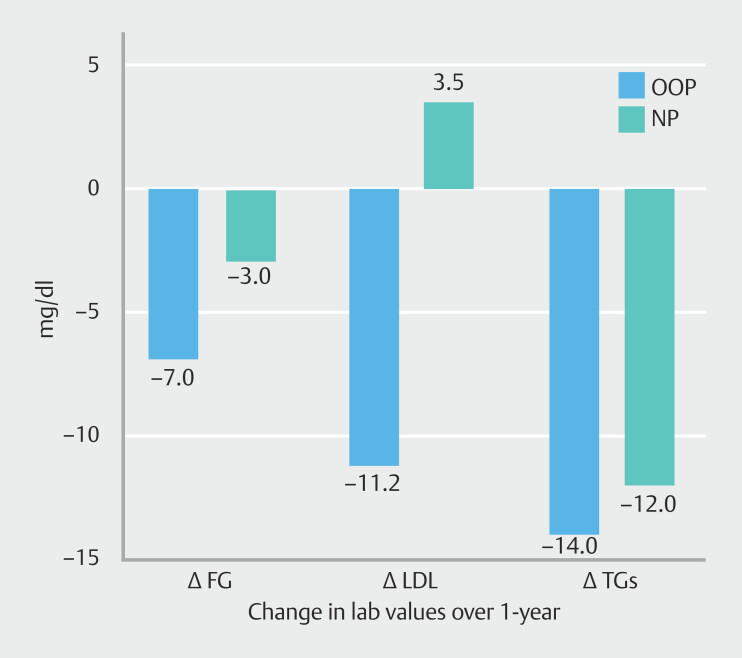
Median differences between 12-month follow-up and baseline values for three laboratory values. Out-of-pocket (OOP) patients were compared with no payment (NP) patients.

**Fig. 4:**
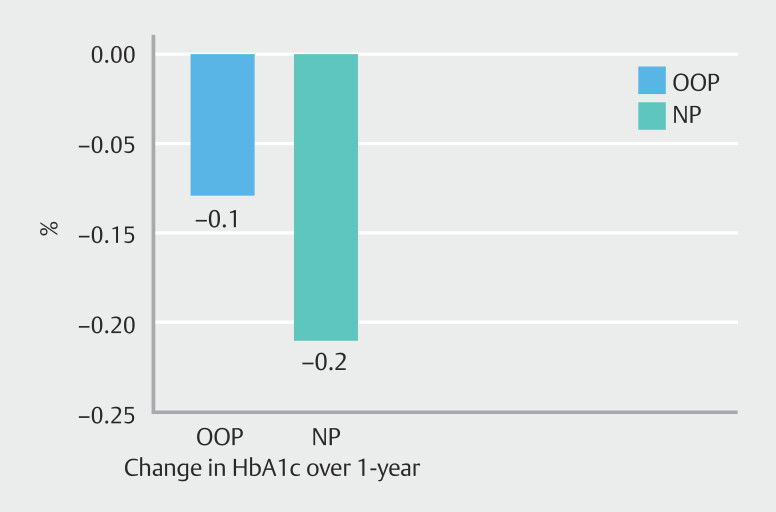
Median differences between 12-month follow-up and baseline values for HbA1c values. Out-of-pocket (OOP) patients were compared with no payment (NP) patients.

FG (n = 101), HbA1c (n = 91), LDL (n = 108), and TG (n = 108) decreased from baseline to follow-up in both NP and OOP groups. The differences between both groups did not appear significant for these parameters ( Table 2 , Fig. 3 , Fig. 4 ).

We examined the effects of group participation (OOP vs. NP) and time (baseline vs. follow-up) on FG, HbA1c, LDL, and TG. The NP group was the reference for between-group comparisons, whereas time of ESG was the reference time point for within-group comparisons.

#### Fasting glucose

For FG, there was no statistically significant difference between the OOP group and the NP group 107.2 (102.1–112.3) vs 112.2 (98.9–125.9), respectively, with between-group difference of 5.0 (–9.5–19.5) at baseline or at 12 months 98.4 (94.5–102.3) vs 101.2 (93.7–108.6), respectively, with between-group difference of 2.8 (-5.6–11.2). Over time, within-group difference was -8.8 (–15.2 to –2.4) for OOP group and -11 (–26.4–4.4) for NP group.

#### HbA1c

For HbA1c, there was no statistically significant difference between the OOP group and the NP group at baseline (5.7 [5.5–5.9]) vs. 5.8 [5.2–5.6]), with a between-group difference of -0.27 (–1.5–1.0). At 12 months, HbA1c levels remained comparable (5.4 [5.3–5.6] vs. 5.4 [5.2–5.6]), with a between-group difference of -0.4 (–2.4–1.5).

Over time, within-group differences were 0.1 (–0.3–0.5) in the OOP group and -0.05 (–2.3–2.2) in the NP group, indicating minimal change in HbA1c within both groups.

#### LDL

For LDL, there was no statistically significant difference between the OOP group and NP group at baseline (110.3 [104.1–116.4]) vs. 110.4 [93.8–127.0]), respectively), with a between-group difference of 0.11 (-17.6–17.8). Similarly, at 12 months, LDL levels remained comparable (99.8 [93.3–106.3] vs. 113.9 [97.0–130.8], respectively), with a between-group difference of 14.1 (–4.0–32.3).

Over time, within-group differences were –10.5 (–28.2–7.2) in the OOP group and 3.5 (–20.2–27.2) in the NP group, suggesting a reduction in LDL in the OOP group but not in the NP group.

#### Triglycerides

For TGs, no statistically significant difference was found between the OOP group and the NP group at baseline (148.9 [137.1–160.8] vs. 128.4 [106.0–150.8], respectively), with a between-group difference of 20.6 (–45.9–4.8). Similarly, at 12 months, TG levels remained similar (126.6 [115.7–137.4] vs. 109.2 [92.6–125.7]), with a between-group difference of –17.4 (–37.2–2.4).

Over time, within-group differences were –22.4 (–38.4 to –6.3) in the OOP group and –19.2 (–47.1–8.7) in the NP group, indicating a reduction in TGs within both groups.

### Treatment adherence

There also appeared to be no significant difference between reported adherence to exercise (total n = 157) at 1 year between the OOP and NP groups (79%, 95% CI 71.9–85.6 vs 92%, 95% CI 76.6–98.3).

## Discussion

Having financial "skin in the game" did not lead to improved weight loss, comorbid condition resolution, nor treatment adherence in our ESG cohort, because no differences were identified between patients with or without OOP costs associated with their ESG. Although seemingly non-significant, there was a trend toward slightly increased weight loss in the NP group as opposed to the OOP group at 6 months, which further refutes the “skin in the game” hypothesis; however, this trend was reversed at 12 and 24 months. There was also no difference in treatment adherence between groups, but rather, a trend toward increased adherence among NP patients. Improvements in comorbidity-related laboratory values were similar between both groups for all lab values. Both groups saw an expected improvement in mean levels of FG, HbA1c, LDL and TGs.

Due to statistical limitations imposed by small sample size, repeated measures with GLM were conducted for the continuous outcomes to evaluate changes both within and between groups. In addition, outcomes were not stratified by sex due to small sample size as well, because meaningful conclusions would be hard to draw by decreasing sample size further. However, sex representation in the overall sample is similar to that previously reported in the literature 6 ; therefore, we suspect our results to be representative of real-world settings. Baseline characteristics were described for both groups. Overall rates of comorbidities and other factors such as age and sex were similar between groups, except for presence of diabetes being considerably higher in the NP group. In addition, to account for inconsistent follow-up and dropout rates, baseline characteristics were also assessed for patients who had specified outcomes of weight at 6, 12, and 24 months and compared with those who did not, and was reported in the supplementary material. Overall differences between groups remained mostly similar, and differences in rates of HTN appeared decreased by 12 and 24 months, potentially due to smaller sample sizes and less power to detect said differences.

Insurance coverage for adult obesity treatment services across Medicaid and state health insurance programs has expanded over the last decade but is still lacking in many states 7 . Despite the growing obesity epidemic, the smallest increase in coverage has been for bariatric surgeries, whereas the largest increase has been for nutritional counseling 7 . We speculate that insurance coverage for non-surgical endoscopic weight loss procedures may also fall short of clinical need, despite robust data showing efficacy and safety for weight loss 2 in patients who might otherwise not be eligible for bariatric surgery 1 . In contrast, pharmacologic therapies for weight loss are slowly being included in health care plans, despite concerns about their high costs 8 . The initial cost of an ESG procedure can vary, and one estimate indicates it is about 16,360 US dollars 9 . However, when comparing ESG to pharmacologic strategies for weight loss, ESG was found to be more cost-effective than a GLP-1 agonist such as semaglutide, even when accounting for those who needed a repeat ESG 9 , and was associated with a reduced total cost of 33,583 US dollars over a 5-year time period. This same study also showed that ESG sustained greater weight loss over 5 years compared with semaglutide (BMI 31.7 vs 33.0) 9 , indicating that coverage of this procedure may not only be indicated, but also economical. In addition, coverage may also affect initial motivation to seek obesity-related treatments. Ard et. al demonstrated that patients with insurance coverage in their cohort were younger and had a lower BMI at baseline, and thus, might be seeking treatment at an earlier stage in obesity 5 . One explanation for this lack of coverage in obesity treatment is that people with obesity are incorrectly perceived as unmotivated and at fault for their weight 10 , and therefore, should take ownership of their treatment. By extension it is also falsely assumed that having to pay for treatment OOP will ensure better outcomes by prompting patients to be more adherent to nutritional and lifestyle recommendations because they have “skin in the game” 5 . In a national poll done of United States adults in 2011, only 55% of respondents endorsed Medicaid coverage for bariatric surgery. Conversely, Medicaid enrollees and low-income respondents had greater odds of endorsing it compared with high-income, private insurance responders 11 .

The burden of shouldering the financial cost of obesity treatment is compounded by the fact that obesity is more prevalent in poverty-dense counties in the United States, which are commonly referred to as “food deserts” for their lack of access to fresh and whole non-processed foods 4 . To add insult to injury, one study by Puhl et. Al showed that people with obesity also suffer from stigma and discrimination and are disadvantaged with regard to employment wages and opportunities, as well as promotions and job termination 10 , which is detrimental in a nation largely dependent on employer-sponsored health care coverage. In another study by Puhl et. al, 54% of responders reported facing stigma from co-workers relating to their weight, and 43% faced stigma from their employers or supervisors 12 . This stigma may also impact health care interactions because patients may feel judged by their health care providers and feel that they are less likely to be treated with respect 13 . This further suggests that societal perception is that individuals with obesity are responsible for their weight, and therefore, responsible for treatment costs, all while being more likely to face discrimination, lower wages, and poverty.

This study has several limitations. Despite mostly similar baseline characteristics between OOP and NP groups, they differed with respect to diabetes, with a higher percentage from the NP group having diabetes compared with the OOP group (28% vs 7%), and correspondingly a higher percentage taking diabetes-related medications (24% vs 8.6%). However, baseline FG and HbA1c were similar between the two groups (98.4 vs 101.2 and 5.4 vs 5.4), so we proceeded with assessing changes in these values. They also differed with respect to age, with the OOP group seemingly younger than the NP group. However, the difference does not seem clinically relevant in our opinion and should not significantly affect outcomes. In fact, it should intuitively favor better outcomes in the OOP group, which was not the case in our results, further suggesting that outcomes are not inherently different between groups. In addition, given sparse insurance coverage for ESG, we could not compare an OOP cohort to an insurance coverage cohort. Instead, the NP cohort was made up of clinical trial participants, whose adherence to treatment and follow-up might be inherently affected by close follow-up from the research team. That said, intense study-related follow up and coaching would favor the opposite assumption of our hypothesis, which might confound our results. However, all patients undergoing ESG at our institution undergo the same education and recommended clinical follow-up regardless of their participation in a research study, and appointments and blood draws also are scheduled in the same way, aside from additional tests that may be completed for the NP group as part of the research protocol. Patients in the OOP group are permitted to schedule their clinical follow-up appointments at different healthcare facilities, which likely accounts for the difference in post-procedure follow-up rates. Finally, conclusions are limited by inherent limitations of a retrospective design.

These results demonstrate that there is no significant difference in weight loss, comorbid condition improvement, or treatment adherence after ESG between patients who pay OOP as compared with those who have no cost associated with their procedure. This reinforces the notion that insurance coverage for proven obesity-related treatments like ESG, should become standard.

## Supplementary Material

Supplementary Material Zusatzmaterial
