# Supplementary material for: Financial buy-in does not affect outcomes of endoscopic sleeve gastroplasty: Retrospective cohort
Source: Endosc Int Open. 2025 Jul 23;13:a26317439. doi: 10.1055/a-2631-7439 (PMC12303024; doi:10.1055/a-2631-7439)
Supplement: Supplementary file 1 — Supplementary Material [file 10-1055-a-2631-7439_26347167.pdf]

Supplementary Table 1 Baseline characteristics for OOP patients and NP patients who had a weight recorded at 6 months.

**Variables**	**OOP (n = 122)**	**NP (n = 25)**
Women	98 (80.3)	20 (80.0)
Age	50.5 (41.6-50.4)	54.1 (43.0-56.9)
HTN (yes)	46 (37.7)	15 (60.0)
T2DM (yes)	8 (6.6)	7 (28.0)
HLD (yes)	55 (45.1)	12 (48.0)
Psychiatric disorder(s) (yes)	42 (34.4)	9 (36.0)
Taking medication for HTN (yes)	43 (35.2)	13 (52.0)
Taking medication for T2DM (yes)	8 (6.6)	6 (24.0)
Taking medication for HLD (yes)	29 (23.8)	8 (32.0)
Taking medication for Psychiatric disorders (yes)	45 (36.9)	10 (40)
HTN on medication (≥ 2)	17 (37.0)	5 (33.3)
T2DM on medication (≥ 2 meds)	2 (25.0)	3 (42.9)
HLD on medication: (yes)	24 (43.6)	7 (58.3)
Psychiatric disorders on medication (≥ 2 meds)	25 (59.5)	3 (33.3)
Baseline weight (kg)	100.0 (91.2-117.4)	99.9 (90.5-107.0)
Baseline BMI (Kg/m^2)	36.2 (32.9-40.3)	35.8 (32.7-37.5)
Baseline FG (mg/dL) (n = 96 and 25)	101.0 (92.0-110.8)	98.0 (92.0-117.8)
Baseline LDL (mg/dL) (n = 100 and 25)	107.0 (87.8-136.5)	101.0 (79.0-144.0)
Baseline TG (mg/dL) (n = 102 and 25)	134.0 (105.8-189.0)	127.0 (81.5-157.5)
Baseline HbA1c (%) (n = 49 and 25)	5.6 (5.2-6.1)	5.4 (5.3-6.7)
Smoking (yes)	3 (2.5)	0 (0)
AOM (yes)	18 (14.8)	1 (4.0)

Results are shown as point estimates (median (IQR)) and corresponding 95% confidence intervals.
AOM, anti-obesity medication; FG, fasting glucose; Hba1c, hemoglobin A1c; HLD, hyperlipidemia; LDL, low-density lipoprotein; meds, medications; NP, no payment; OOP, out of pocket; Q1, quartile 1; Q3, quartile 3; T2DM, type 2 diabetes mellitus; TG, triglyceride.

Supplementary Table 2 Baseline characteristics for OOP patients and NP patients who had a weight recorded at 12 months.

Variables	OOP (n = 86)	NP (n = 23)
Women	69 (80.2)	18 (78.3)
Age	50.3 (40.1-57.4)	54.1 (44.4-57.1)
HTN (yes)	34 (39.5)	14 (60.9)
T2DM (yes)	8 (9.3)	7 (30.4)
HLD (yes)	40 (46.5)	11 (47.8)
Psychiatric disorder(s) (yes)	29 (33.7)	9 (39.1)
Taking medication for HTN (yes)	313 (36.0)	12 (52.2)
Taking medication for T2DM (yes)	9 (10.5)	6 (26.1)
Taking medication for HLD (yes)	22 (25.6)	7 (30.4)
Taking medication for Psychiatric disorders (yes)	29 (33.7)	10 (43.5)
HTN on medication (≥ 2)	13 (38.2)	4 (28.6)
T2DM on medication (≥ 2 meds)	3 (37.5)	3 (42.9)
HLD on medication: (yes)	19 (47.5)	6 (54.5)
Psychiatric disorders on medication (≥ 2 meds)	17 (58.6)	3 (33.3)
Baseline weight (kg)	97.5 (88.0-115.5)	99.9 (90.1-107.9)
Baseline BMI (kg/m^2)	35.8 (33.2-40.2)	34.6 (32.7-37.6)
Baseline FG (mg/dL) (n = 68 and 23)	103.5 (92.3-111.8)	98.0 (92.0-118.0)
Baseline LDL (mg/dL) (n = 72 and 23)	108.0 (87.8-135.0)	101.0 (78.0-146.0)
Baseline TG (mg/dL) (n = 72 and 23)	133.5 (107.5-186.8)	113.0 (71.0-157.0)
Baseline HbA1c (%) (n = 36 and 23)	5.5 (5.2-6.1)	5.4 (5.3-6.7)
Smoking (yes)	2 (2.3)	0 (0.0)
AOM (yes)	13 (15.1)	1 (4.3)

Results are shown as point estimates (median (IQR)) and corresponding 95% confidence intervals.
AOM, anti-obesity medication; FG, fasting glucose; Hba1c, hemoglobin A1c; HLD, hyperlipidemia; LDL, low-density lipoprotein; meds, medications; NP, no payment; OOP, out of pocket; Q1, quartile 1; Q3, quartile 3; T2DM, type 2 diabetes mellitus; TG, triglyceride.

Supplementary Table 3 Baseline characteristics for OOP patients and NP patients who had a weight recorded at 24 months.

	OOP (n = 51)	95% CI	NP (n = 20)	95% CI
Women	43 (84.3)	72.6-92.3	16 (80.0)	59.2-92.8
Age	50.1 (42.4-60.0)	47.4-54.1	54.2 (42.2-57.0)	44.9-56.7
HTN (yes)	19 (37.3)	25.0-50.9	12 (60.0)	38.4-78.9
T2DM (yes)	7 (13.7)	6.4-25.1	5 (25.0)	10.2-46.4
HLD (yes)	24 (47.1)	33.8-60.6	9 (45.0)	25.1-66.2
Psychiatric disorder(s) (yes)	21 (41.2)	28.5-54.9	8 (40.0)	21.1-61.6
Taking medication for HTN (yes)	18 (35.3)	23.3-48.9	11 (55.0)	33.8-74.9
Taking medication for T2DM (yes)	7 (13.7)	6.4-25.1	5 (25.0)	10.2-46.4
Taking medication for HLD (yes)	15 (29.4)	18.3-42.8	7 (35.0)	17.2-56.8
Taking medication for Psychiatric disorders (yes)	23 (45.1)	32.0-58.7	8 (40.0)	21.1-61.6
HTN on medication (≥ 2)	7 (36.8)	18.2-59.1	4 (33.3)	12.5-61.2
T2DM on medication (≥ 2 meds)	2 (28.6)	6.5-64.8	3 (60.0)	20.9-90.6
HLD on medication: (yes)	13 (54.2)	34.7-72.7	6 (66.7)	34.8-89.6
Psychiatric disorders on medication (≥ 2 meds)	14 (66.7)	45.4-83.7	3 (37.5)	11.9-70.5
Baseline weight (kg)	96.9 (87.0-110.0)	93.2-103.0	99.9 (90.0-108.6)	90.1-104.9
Baseline BMI (kg/m^2)	35.1 (33.1-39.4)	34.1-36.9	35.8 (32.8-37.3)	33.5-36.6
Baseline FG (mg/dL) (n = 39 and 20)	100.0 (91.0-110.0)	98.0-100.0	97.5 (91.3-117.8)	92.0-117.0
Baseline LDL (mg/dL) (n = 41 and 20)	96.0 (82.0.-136.5)	91.0-120.5	92.0 (78.0-117.5)	79.0-116.0
Baseline TG (mg/dL) (n = 41 and 20)	127.0 (100.0-194.0)	115.5-164.0	120.0 (71.0-156.8)	91.0-154.0
Baseline HbA1c (%) (n = 20 and 20)	5.6 (5.2-6.3)	5.0-5.6	5.5 (5.3-6.8)	5.3-6.6
Smoking (yes)	2 (3.9)	0.8-12.0	0 (0.0)	N/A
AOM (yes)	6 (11.8)	5.1-22.7	0 (0.0)	N/A

Results are shown as point estimates (median (IQR)) and corresponding 95% confidence intervals.

AOM, anti-obesity medication; CI, confidence interval; FG, fasting glucose; Hba1c, hemoglobin A1c; HLD, hyperlipidemia; LDL, low-density lipoprotein; meds, medications; NP, no payment; OOP, out of pocket; Q1, quartile 1; Q3, quartile 3; T2DM, type 2 diabetes mellitus; TG, triglyceride.
